# The Cross‐Sectional Areas and Anterior–Posterior Balance of the Cervical Paraspinal Muscles in Dropped Head Syndrome and Cervical Spondylotic Myelopathy: A Propensity Score‐Matched Analysis

**DOI:** 10.1002/jsp2.70047

**Published:** 2025-01-30

**Authors:** Takashi Sono, Kenta Ijiri, Kensaku Kakehi, Soichiro Masuda, Takayoshi Shimizu, Koichi Murata, Shuichi Matsuda, Bungo Otsuki

**Affiliations:** ^1^ Department of Orthopaedic Surgery Kyoto University Graduate School of Medicine Kyoto Japan

**Keywords:** anterior–posterior muscle balance, Dropped head syndrome, longus colli muscle, semispinalis cervicis muscle

## Abstract

**Introduction:**

Dropped head syndrome (DHS) is characterized by weakness of the neck extensor muscles. However, few studies have assessed the cross‐sectional areas (CSAs) of the cervical paraspinal muscles (CPM) and their anterior–posterior balance in DHS. This study aimed to elucidate the pathognomonic findings of DHS by comparing the CSAs and anterior–posterior balance of the CPM in patients with DHS and cervical spondylotic myelopathy (CSM), using magnetic resonance imaging (MRI).

**Methods:**

We compared the CSAs and anterior–posterior balance of the CPM in patients with DHS and CSM using MRI. Patients with CSM were selected in an age‐ and sex‐matched manner, using the propensity score. The longus colli (LC) muscle was selected as the anterior muscle; and the semispinalis cervicis (SSC), splenius capitis (SC), and multifidus muscles (MM) were selected as the posterior muscles. We calculated LC/SSC, LC/SC, LC/MM, and LC/(SSC + SC + MM), as indicators of neck muscle balance.

**Results:**

The DHS and the CSM cohort comprised 26 and 52 patients, respectively. Both cohorts had a mean age of 71‐year‐old. There were no significant differences in the CSAs and most of the indicators of neck balance between the two cohorts. However, the LC/SSC was significantly higher in the DHS cohort than that in the CSM cohort (40.3% and 29.1%, respectively; *p* < 0.01).

**Conclusions:**

Our study highlights a unique anterior–posterior imbalance in the CPM of DHS patients, differing from CSM patients. Strengthening the SSC muscle could be a key to preventing DHS progression.

## Graphical abstract

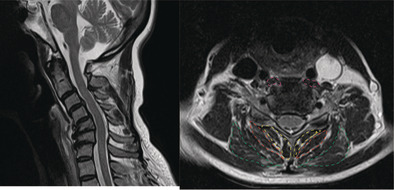

## 1 Introduction

Dropped head syndrome (DHS) is a clinical condition characterized by a profound weakness of the neck extensor muscles, leading to an inability to hold the head up and a resultant chin‐on‐chest deformity [1, 2]. It can considerably affect a patient's quality of life, as patients with DHS frequently experience difficulty in performing activities of daily living. There are numerous etiologies of DHS, including Parkinson's disease and related neurological syndromes; muscular diseases such as myositis and endocrine myopathies; neuromuscular diseases, including myasthenia gravis, motor neuron diseases, chronic inflammatory demyelinating polyneuropathy; idiopathic etiologies; and as a complication of neck irradiation [3, 4, 5, 6, 7].

DHS involves a heterogeneous population; however, few studies have evaluated the correlation between the CSAs of the cervical paraspinal muscles and this condition. Moreover, no study has previously reported on the anterior–posterior balance of the cervical paraspinal muscles. The previous radiological study has compared DHS with cervical spondylotic myelopathy (CSM), using CSM as a control group [8]. We adopted the methodology from this study for our research.

Therefore, in this study, we compared the cross‐sectional areas (CSAs) and anterior–posterior balance of the cervical paraspinal muscles in patients with DHS and CSM, using magnetic resonance imaging (MRI).

## 2 Materials and Methods

### 2.1 Ethical Approval

This study was approved by the Institutional Review Board (IRB) before data collection and analysis (R2901) and followed the Declaration of Helsinki.

### 2.2 Study Population and Design

In this retrospective cohort study, the data of patients with DHS were selected and extracted from the clinical records of our institution between April 2017 and March 2023. Patients with DHS were defined as those with a chin‐on‐chest deformity who could not maintain a horizontal gaze with their necks in an upright position. The data from patients with a known medical history of infection, tumors, ankylosing spondylitis, previous cervical spine surgery, and from patients who had not undergone an MRI were excluded from the statistical analysis. The data of participants with a past medical history of Parkinson's disease; Parkinsonism; myopathies, such as hypothyroid myopathies and those secondary to autoimmune diseases; patients with neuromuscular and motor neuron diseases, as well as polyneuropathies and neck irradiation were included. In addition, during this period, the data from patients with CSM were selected and extracted in the same manner, and an age‐ and sex‐matched 1:2 cohort was created using propensity score (PS) matching.

### 2.3 Imaging

**FIGURE 1:**
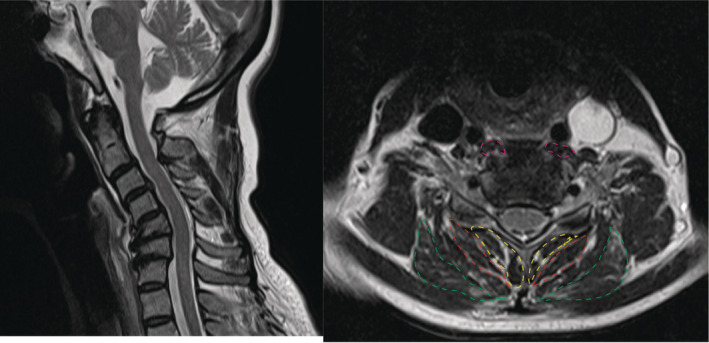
Magnetic resonance images depicting dropped head syndrome. Left and right images reveal the transverse relaxation time (T2)‐weighted sagittal, and axial images of the cervical spine (fifth/sixth cervical vertebral [C5/6] disc level), respectively. Magenta, red, green, and yellow, depict the longus colli, semispinalis cervicis, splenius capitis, and multifidus muscles, respectively.

**FIGURE 2:**
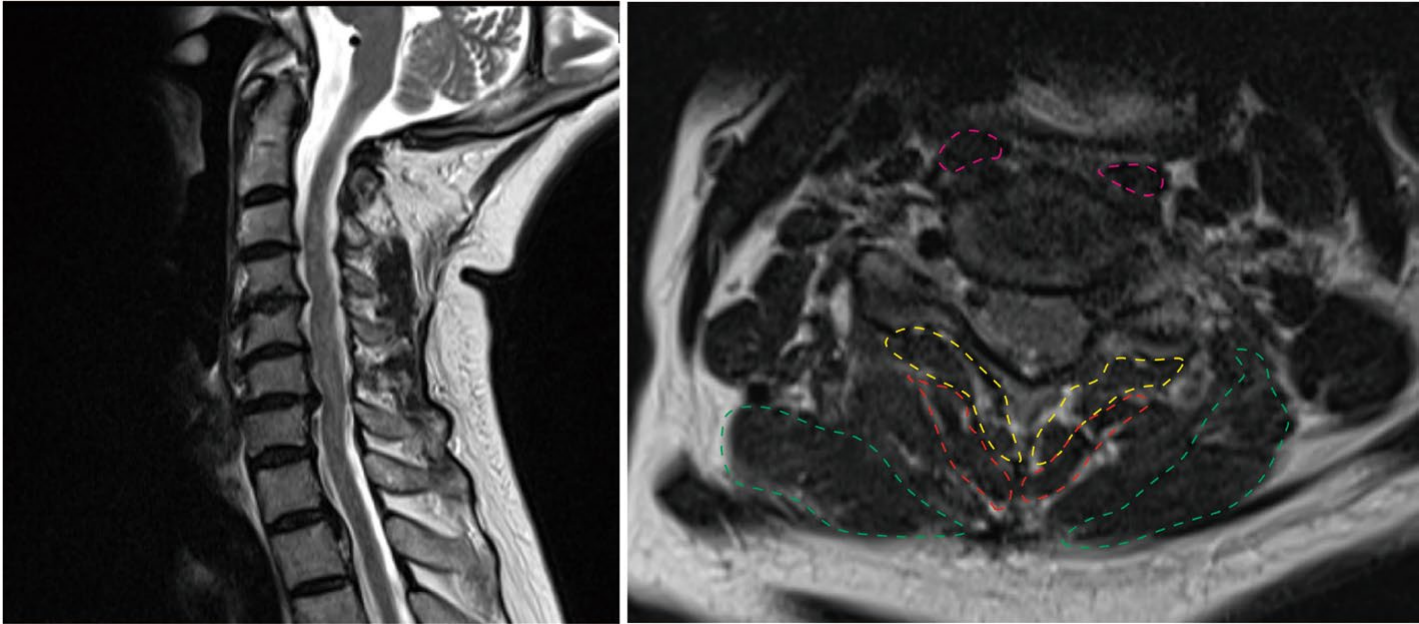
Magnetic resonance images depicting cervical spondylotic myelopathy. Left and right images show the transverse relaxation time (T2)‐weighted sagittal, and axial images of the cervical spine (fifth/sixth cervical vertebral [C5/6] disc level), respectively. Magenta, red, green, and yellow, depict the longus colli, semispinalis cervicis, splenius capitis, and multifidus muscles, respectively.

MR transverse relaxation time (T2)‐weighted images at the fifth/sixth cervical (C5/6) vertebral disc‐level of the paraspinal muscles were obtained, and the CSAs of these muscles were measured using the Centricity Universal Viewer Zero Footprint (GE Healthcare, Milwaukee, WI, USA) (Figures 1 and 2). The longus colli (LC) muscle was selected as the anterior muscle, because it is directly attached to the anterior of the vertebral bodies. The semispinalis cervicis (SSC), splenius capitis (SC), and multifidus muscles (MM) were selected as posterior muscles, due to their attachment to both the lamina and nuchal ligament of the vertebrae, thus effecting neck extension. We compared the muscles bilaterally and chose the larger muscle for the measurement of the CSA. One surgeon performed the measurements thrice, and the mean value was calculated. We selected the C5/6 level to allow for reliable and reproducible assessment of CSA and fatty infiltration at the midportion of the longus colli and multifidus muscle bellies following the previous study [9]. To capture changes in CSA at other levels, evaluations were also conducted at the C6/7 and C7/Th1 levels. We calculated LC/SSC, LC/SC, LC/MM, and LC/(SSC + SC + MM) as indicators of neck balance. Fat infiltration into the posterior cervical paraspinal muscles was evaluated according to the Goutallier classification [10, 11]. The C2–7 angle was measured on a lateral radiograph of the cervical spine to assess cervical lordosis.

### 2.4 Statistical Analysis

PS matching was used to adjust for confounding variables [12]. The PS for DHS was estimated using a logistic regression model that included the demographics of age and sex.

PS matched analysis with a ratio of 1:2 was performed when comparing participants who had DHS with those who had CSM. Nearest neighbor matching within a caliper width equal to 0.2 of the standard deviation of the PS logit without replacement was performed. A standardized mean difference of less than 0.1 indicated a good balance between the two cohorts [13]. The PS analyses were conducted using R (version 4.2.1, R Foundation for Statistical Computing, Vienna, Austria). The remaining analyses were performed using JMP Pro (Version 13.0, SAS Institute Inc., Cary, NC, USA). Dunnett's test was used to analyze the radiographic, MRI, and baseline demographic results of the participants. Statistical significance was set at *p* < 0.05.

## 3 Results

### 3.1 Baseline Demographic Results

The DHS cohort consisted of 26 patients, of which six were male and 20 were female. The CSM cohort consisted of 52 patients, of which 12 were male and 40 were female. Both cohorts had a mean age of 71‐years‐old. The two cohorts were matched 1:2 for age and sex, using the PS matching method (Table 1). Regarding the past medical history, the incidence of Parkinson's disease and parkinsonism was statistically significantly greater in the DHS cohort than in the CSM cohort (*p* < 0.01) (Table 1). There were no statistically significant differences in the incidence of myopathies between the two cohorts (*p* = 0.74) (Table 1). There were no patients with neuromuscular diseases, motor neuron diseases, polyneuropathies, or neck irradiation. Lateral radiographs revealed that the cervical spine (C2–7) angle was statistically significantly smaller in the DHS cohort than in the CSM cohort (*p* < 0.001) (Table 1).

**TABLE 1:** Baseline demographics of the participants.

	DHS	CSM	SMD
Age (mean ± SD)	71.3 ± 0.6	71.0 ± 10.0	0.03
Sex (female: male)	20: 6	40: 12	< 0.001
Past medical history			***p***
*Parkinson's disease and parkinsonism*	5	1	< 0.01*
*Myopathies (hypothyroid myopathies and myopathies secondary to autoimmune diseases)*	2	3	0.74
Radiographs			***p***
C2–7 angle (°)	−14.8 ± 28.6	8.8 ± 14.2	< 0.001**

Abbreviations: C2–7 angle, cervical spine angle; CSM, Cervical spondylotic myelopathy; DHS, Dropped head syndrome; SMD, Standardized mean difference.

* *p* < 0.01.

** *p* < 0.001.

### 3.2 The Cross‐Sectional Areas of the Cervical Paraspinal Muscles and Goutallier Classification

There were no statistically significant differences in the degree of fat infiltration between the two cohorts, as per the Goutallier classification (*p* = 0.19) (Table 2).

**TABLE 2:** Cross‐sectional areas of the cervical paraspinal muscles (means ± standard deviations; cm^2^) and Goutallier classification at the C5/6 level.

	DHS	CSM	*p*
Multifidus muscle (MM)	0.76 ± 0.35	0.71 ± 0.27	0.44
Semispinalis cervicis (SSC) muscle	0.94 ± 0.43	1.07 ± 0.41	0.17
Splenius capitis (SC) muscle	1.75 ± 0.78	1.51 ± 0.54	0.12
Longus colli (LC) muscle	0.29 ± 0.09	0.28 ± 0.10	0.47
SC+ SSC + MM	3.45 ± 1.26	3.29 ± 0.99	0.55
Goutallier classification	1.42 ± 1.23	1.75 ± 0.92	0.19

Abbreviations: CSM, Cervical spondylotic myelopathy; DHS, Dropped head syndrome.

Regarding the CSAs of the cervical paraspinal muscles, the average of CSAs of the LC muscles was 0.29 and 0.28 cm^2^, in the DHS and CSM cohorts, respectively. The CSAs of the SC muscles were 1.75 and 1.51 cm^2^, in the DHS and CSM cohorts, respectively. The CSAs of the SSC muscles were 0.94 and 1.07 cm^2^, in the DHS and CSM cohorts, respectively. The CSAs of the MMs were 0.76 and 0.71 cm^2^, in the DHS and CSM cohorts, respectively. There were no statistically significant differences in the CSAs between the two cohorts for any of the muscles (Table 2). At C6/7 and C7/Th1 levels, there were no statistically significant differences in the CSAs between the two cohorts for any of the muscles (Table S1).

### 3.3 The Anterior–Posterior Balance of the Cervical Paraspinal Muscles

Additionally, the percentage values of LC/(SC + SSC + MM) were 10.3% and 8.94%, in the DHS and CSM cohorts, respectively (Table 3).

**TABLE 3:** The anterior–posterior balance of the cervical paraspinal muscles (means ± standard deviations; %) at the C5/6 level.

	DHS	CSM	*p*
LC/(SC + SSC + MM)	10.3 ± 6.0	8.94 ± 3.38	0.21
LC/SC	22.9 ± 17.5	20.1 ± 8.51	0.35
LC/SSC	40.3 ± 25.0	29.1 ± 14.0	0.01*
LC/MM	46.8 ± 25.6	43.9 ± 20.5	0.58

Abbreviations: LC, Longus colli; MM, Multifidus muscles; SC, Splenius capitis; SSC, Semispinalis cervicis.

* *p* < 0.05.

**FIGURE 3:**
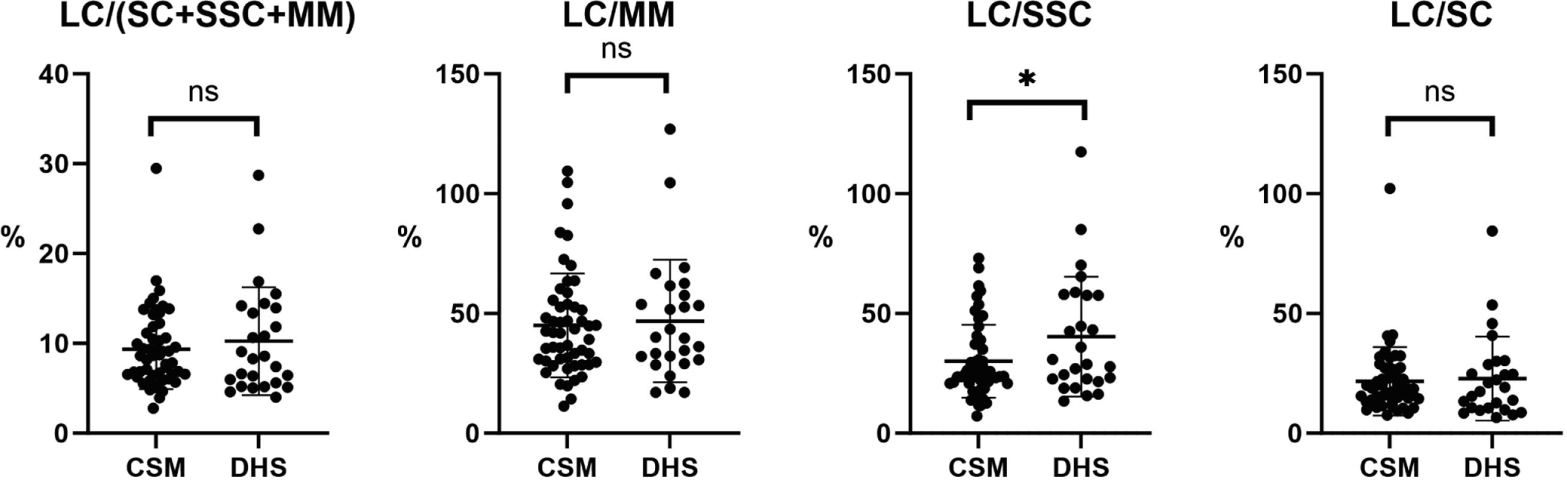
Graphs showing the anterior–posterior balance of the cervical paraspinal muscles, using means and standard deviations. Each dot reveals the individual parameters: Cervical spondylotic myelopathy (CSM); and dropped head syndrome (DHS). LC, Longus colli; SSC, Semispinalis cervicis; SC, Splenius capitis; and MM, Multifidus muscles; NS, Not significant; *p < 0.05.

The percentage values of LC/MM were 46.8% and 43.9%, in the DHS and CSM cohorts, respectively. The percentage values of LC/SC were 22.9% and 20.1%, in the DHS and CSM cohorts, respectively. Thus, there were largely not any statistically significantly differences in the ratios and percentage values of the indicators of neck balance between the two cohorts. However, the LC/SSC percentage value was statistically significantly higher in the DHS cohort than that in the CSM cohort (40.3% and 29.1%, respectively; *p* < 0.01) (Table 3) (Figure 3). At C6/7 and C7/Th1 levels, there were no statistically significant differences in the anterior–posterior muscle balance between the two cohorts. (Table S2).

## 4 Discussion

DHS is a condition that involves the neck extensor muscles; however, in this study, there were no statistically significant differences in the CSAs between the two cohorts for any of these muscles. Nevertheless, the LC/SSC ratio was statistically significantly higher in the DHS cohort than that of the CSM cohort. These results suggested that the anteroposterior balance of the cervical paraspinal muscles differed between the DHS and CSM cohorts. Anteriorly biased anteroposterior balance may make cervical extension difficult.

The MM muscle originates from the articular process and inserts on the spinal process two or three levels below. The SC muscle originates from the lower half of the nuchal ligament and the spinous processes of the 7th cervical vertebra and the superior 3 thoracic vertebrae. It inserts onto the mastoid process of the temporal bone. The SSC muscle originates from the transverse processes of the first to seventh thoracic (T1–7) vertebrae, runs obliquely superiorly, and attaches to the spinous processes of the C2–7 vertebrae [14]. The MM muscle attributes to the neck stability and rotation and the SC muscle attributes to the neck extension, rotation and lateral flexion and the SSC muscle attributes to the neck extension [15].

The importance of the SSC muscle has long been documented, and it has been found that in patients with CSM, the preoperative CSA of this muscle is a risk factor for the postoperative loss of cervical lordosis after laminoplasty [16]. Additionally, SSC sarcopenia is correlated with deteriorating cervical sagittal balance and junctional alignment subsequent to posterior cervical fusion surgery for myelopathy [17]. Moreover, a correlation exists between the CSAs of deep paraspinal muscles and bone union after anterior cervical decompression and fusion [18]. Particularly, it has been shown that the CSAs of the extensor muscles exhibit a significant negative correlation with the fusion time. However, no study has formerly reported on the anterior–posterior balance of the cervical paraspinal muscles, and this study is novel in this respect.

MRI performed in the supine position is a very useful modality for assessing the cervical paraspinal muscles in patients with DHS whose cervical spine alignment substantially changes, depending on their posture [19].

Regarding the conservative treatment of DHS, it is important to first treat the underlying diseases, such as Parkinson's disease and myopathies. Second, it is essential to strengthen the SSC muscles, through substantiated neck exercises [20, 21, 22]. As a home exercise, we recommend an exercise where the chin is supported by the hand, similar to resting the chin on the hand, and then lifting the chin by activating the neck extensor muscles including SSC muscles.

Regarding surgical treatment, posterior or anterior–posterior fixation is commonly performed for DHS, depending on various parameters, including the apex of cervical kyphosis, degree of degeneration, T1 slope, and status of bony fusion of the cervical spine [23, 24]. Based on the impact of the SSC muscles on DHS, the range of fusion should be where the SSC muscles are present, from C2 to T7. However, fixation down to T1 or T5 can provide sufficient stability [25]. When the deformity is hard‐in‐consistency and accompanied by bony fusion, an additional osteotomy is necessary. Moreover, when the thoracolumbar spine is unbalanced with a T1 slope ≥ 40°or positive sagittal vertical axis, corrective thoracolumbar deformity surgery should first be performed [26].

This study has three limitations. First, the CSAs of the muscles were not adjusted for by body mass index. Muscle area changes with body size [27]; however, corrections were not performed in this study. Second, this study did not evaluate cervical paraspinal muscle quality. Endo et al. reported necrosis, microvessel proliferation, and atrophy of the neck extensor muscles and ligament degeneration in patients with DHS [28]. For the most part, the MRI findings of the cervical paraspinal muscles were not statistically significantly different; nonetheless, the differences in muscle quality between participants with DHS and those with CSM may have existed. Third, regarding underlying conditions, DHS as a heterogeneous population was not matched with that of CSM. Finally, whole spine sagittal alignment is not evaluated. The previous report indicated a negative correlation between total body muscle mass and Pelvic Tilt in patients with cervical degenerative disease [29]. In the future, studies on DHS patients should not only focus on cervical paraspinal muscles but also include total body muscle mass, T1 slope, and sagittal alignment.

In conclusion, the anterior–posterior balance of the cervical paraspinal muscles in participants with DHS was different from that in participants with CSM. There were no statistically significant differences in the CSAs between the two cohorts for any of the muscles. These findings may lead to better identification of and awareness about the numerous underlying etiologies of DHS, as well as improved prevention of its progression by strengthening the SSC muscle.

## Conflicts of Interest

The authors declare no conflicts of interest.

## Supporting information


**Table S1.** Cross‐sectional areas of the cervical paraspinal muscles (means ± standard deviations; cm^2^) at C6/7 and C7/Th1 levels.
**Table S2.** The anterior–posterior balance of the cervical paraspinal muscles (means ± standard deviations; %) at C6/7 and C7/Th1 levels.
