# Supplementary material for: The Cross‐Sectional Areas and Anterior–Posterior Balance of the Cervical Paraspinal Muscles in Dropped Head Syndrome and Cervical Spondylotic Myelopathy: A Propensity Score‐Matched Analysis
Source: JOR Spine. 2025 Jan 30;8(1):e70047. doi: 10.1002/jsp2.70047 (PMC11782067; doi:10.1002/jsp2.70047)
Supplement: Supplementary file 1 — Table S1. Cross‐sectional areas of the cervical paraspinal muscles (means ± standard deviations; cm2) at C6/7 and C7/Th1 levels. Table S2. The anterior–posterior balance of the cervical paraspinal muscles (means ± standard deviations; %) at C6/7 and C7/Th1 levels. [file JSP2-8-e70047-s001.docx]

***Supplementary Table 1: Cross-Sectional Areas of the Cervical Paraspinal Muscles (Means ± Standard Deviations; cm^2^) at C6/7 and C7/Th1 levels.***

| Level | Muscle | DHS | CSM | p-value |
| --- | --- | --- | --- | --- |
| C6/7 | Multifidus (MF) | 1.20±0.81 | 1.22±0.65 | 0.92 |
|  | Semispinalis (SS) | 1.74±0.67 | 1.82±0.45 | 0.53 |
|  | Splenius (Sp) | 2.06±1.01 | 1.78±0.59 | 0.12 |
|  | Longus Coli (LC) | 0.59±0.17 | 0.63±0.22 | 0.34 |
|  | MF+SS+SP | 5.00±1.97 | 4.76±1.27 | 0.52 |
| C7/Th1 | Multifidus (MF) | 0.96±0.62 | 0.87±0.38 | 0.42 |
|  | Semispinalis (SS) | 1.52±0.60 | 1.60±0.44 | 0.49 |
|  | Splenius (Sp) | 1.70±0.93 | 1.53±0.50 | 0.29 |
|  | Longus Coli (LC) | 0.58±0.21 | 0.60±0.21 | 0.80 |
|  | MF+SS+SP | 4.18±1.50 | 4.00±0.86 | 0.50 |

***Supplementary Table 2: The Anterior-Posterior Balance of the Cervical Paraspinal Muscles (Means ± Standard Deviations; %) at C6/7 and C7/Th1 levels.***

| Level | Muscle | DHS | CSM | p-value |
| --- | --- | --- | --- | --- |
| C6/7 | LC/MF+SS+SP | 13.4±8.02 | 14.3±5.38 | 0.57 |
|  | LC/MF | 71.0±51.2 | 62.8±32.3 | 0.39 |
|  | LC/SS | 39.5±19.2 | 36.3±12.9 | 0.37 |
|  | LC/SP | 36.5±22.4 | 38.1±14.9 | 0.71 |
| C7/Th1 | LC/MF+SS+SP | 15.1±6.22 | 15.0±4.56 | 0.96 |
|  | LC/MF | 80.0±44.5 | 75.4±27.8 | 0.57 |
|  | LC/SS | 44.2±25.8 | 38.9±15.6 | 0.26 |
|  | LC/SP | 44.7±30.6 | 44.1±27.1 | 0.92 |

LC, longus colli; SC, splenius capitis; SSC, semispinalis cervicis and MM, multifidus muscles
